# Erythropoietin Administration for Anemia Due to Chronic Kidney Disease - Subcutaneous OR Intravenous, What Do We Know So Far?

**DOI:** 10.7759/cureus.10358

**Published:** 2020-09-10

**Authors:** Muhammad Hasan Shahab, Shahzeen Saifullah Khan

**Affiliations:** 1 Internal Medicine, Memon Medical Institute Hospital, Karachi, PAK; 2 Paediatrics, Dr. Ruth K.M. Pfau Civil Hospital Karachi / Dow Medical College, Karachi, PAK; 3 Accident and Emergency, Memon Medical Institute Hospital, Karachi, PAK

**Keywords:** chronic kidney disease, esrd, subcutaneous, intravenous, erythropoietin, dialysis, cost, efficacy, frequency, anemia

## Abstract

The prevalence of anemia in chronic kidney disease (CKD) patients is almost twice that of the normal population and its severity increases exponentially as the disease worsens, dramatically affecting the quality of an individual’s life. The advent of erythropoiesis stimulating agents (ESA) in the 1980s saw a revolutionary change in the treatment of anemia in CKD patients, drastically improving quality of life (QoL), overall health and reducing the need for blood transfusions. Numerous ESAs have been developed ever since and are in current use, with the primary routes of administration being intravenous (IV) and subcutaneous (SC) injections. Their use, however, has stirred significant controversy over the last two decades. Additionally, despite numerous studies and trials, the latest international recommendations for their use do not provide clear cut guidance with well-grounded evidence on the recommended route of administration for different sets of patients. Instead, this decision has mainly been left up to the physician’s discretion, whilst keeping certain key factors in mind. This review shall summarize, discuss and compare the findings of previous studies on various factors governing the two aforementioned routes of administration and identify areas that need further exploration.

## Introduction and background

Anemia, defined as serum haemoglobin (Hb) levels ≤12 gm/dL in women and ≤13 gm/dL in men, is a common complication of chronic kidney disease (CKD), being prevalent twice as much in the affected adult population (15.4%) as opposed to the general population (7.4%). The prevalence of anemia tends to be correlated with the severity of underlying kidney disease, prevailing in 8.1% of patients with stage 1 CKD to 53.4% in stage 5 CKD [1]. It is frequently associated with a poorer quality of life (QoL) that deteriorates further as the disease progresses, and commonly results in cognitive impairment, reduced exercise capacity, worsening of cardiac function, increased cardiovascular morbidity and ultimately contributes to an increased overall mortality rate [1,2].

Poor kidney function and accumulation of uremic toxins is a known cause of anemia in CKD patients in addition to reduced erythropoietin (EPO) production due to loss of functioning renal parenchyma, hyporesponsiveness to EPO, iron deficiency, chronic inflammation, and shortened red blood cell survival [1-3].

If left untreated, it has a significant detrimental impact on the patient’s QoL, their overall health and healthcare costs. A study in pre-dialysis CKD patients found a significant increase (p < 0.0001) in monthly treatment costs between CKD patients who had untreated anemia against those without anemia. In a similar study involving patients with end-stage renal disease (ESRD), who were undergoing dialysis, medical costs were found to be 8.9% higher for every month of treatment when Serum Hb was less than 11 gm/dL [2].

While the treatment of anemia in CKD involves managing multiple causative factors as highlighted above, the most significant factor is the deficiency of EPO. As a result, the use of erythropoiesis stimulating agents (ESAs) since 1989 has significantly improved the management of anemia [4]. The need for blood transfusions in CKD patients has gone down significantly, whilst improving QoL and exercise capacity [5,6]. A study conducted in the United States in 2005 indicated almost 99% of in-center haemodialysis (HD) patients receiving ESA. The magnitude of the revolutionary change brought by these agents can also be estimated from the fact that in 2004, EPO therapy totaled $1.8 billion - the single largest Medicare drug expenditure in the United States [4,7].

The 2012 Kidney Disease Improving Global Outcomes (KDIGO) guidelines regarding anemia in CKD present evidence and recommendations of varying strength. With regards to ESA therapy in adult CKD patients, they strongly recommend against intentionally increasing Hb > 13.0 gm/dl, backed by high quality evidence. They suggest that ESA therapy should be initiated in ESRD patients when Hb is between 9-10.0 gm/dl with an aim to keep it above 10 gm/dl but not above 11.5 gm/dl, backed by low to moderate quality evidence [8]. In patients with non-dialysis dependent, advanced CKD with Hb < 10.0 gm/dl, they suggest that the decision to treat using ESAs should be weighed against various other factors but this is supported by low quality evidence. These guidelines however, exhibit low to very low quality evidence when it comes to recommendations about the type of ESA, dosing frequency, and their route of administration, the last of which is the main focus of this review [8].

Since their inception, ESAs have undergone significant development and innovation, in line with associated healthcare costs, pharmacokinetics, drug efficacy, side effect profiles and dosing frequency as well as the route of administration. The ones commonly used include the first generation erythropoietin-alfa and beta and the second generation agent Darbepoetin [4]. Their use, however, has come under serious scrutiny over the last two decades owing to certain studies that showed an increase in overall mortality and adverse cardiovascular as well as cerebrovascular events [9].

Target Hb levels secondary to ESA therapy remain a matter of controversy worldwide, with higher Hb targets (>11.0 gm/dl) being tied to an increased risk of adverse events and no significant improvement in QoL against that of partially corrected anemia, i.e., Hb (9.5 - 11.0) gm/dl [8,10]. One rare outcome that gained significant attention was that of ESA-induced pure red cell aplasia (PRCA) between 1998 and 2004, mainly associated with the subcutaneous (SC) route of administration, and the exact cause of which is thought to be the production of anti-EPO antibodies. However, this was found to be associated with one specific product of epoetin-alfa “Eprex/Erypo” in Europe and was not associated at the same scale with other subcutaneously administered epoetin products. This was appropriately dealt with by the formulation of ESAs with a new structure [11,12].

## Review

Method

For this review, we performed a literature search on the PubMed database (1988 - Present date) using combinations of multiple keywords and Medical Subject Heading (MeSH) terms - anemia, ESRD, erythropoietin, dialysis, chronic kidney disease, patient satisfaction, etc. The study types that were reviewed included review articles, meta-analyses, systematic reviews, multicenter studies, randomized clinical trials, excerpts from various journals and documents, and clinical trials. To meet the criteria for inclusion, articles also needed to involve human subjects only and be published in English. Case reports, editorials, commentaries, opinions and animal studies were not included in this review. The most important point for inclusion was individual article relevance to the scope of this review. However, this was carried out manually after screening articles on the basis of their titles and abstracts.

Results

Using the aforementioned searching strategy, we found a total of 41,271 articles, of which 35,419 were found to be duplicated (see Table 1). A total of 5,852 articles were therefore found after the initial search.

**Table 1:** Results of the initial literature search, carried out on 15th August 2020, on the PubMed Database using combinations of different MeSH terms and regular keywords ESRD: End-stage renal disease; MeSH: Medical Subject Heading

Search number	Query		Results
1	Erythropoietin AND Quality Of Life		1,302
2	Erythropoietin AND ESRD		3,519
3	Erythropoietin AND Safety AND ESRD		229
4	Erythropoietin AND Pure Cell Red Aplasia		272
5	ESRD AND Pure Cell Aplasia		86
6	Erythropoietin AND Cost AND Subcutaneous		109
7	Anemia AND Chronic Kidney Disease		7,353
8	Anemia AND ESRD		5,322
9	Erythropoietin AND Anemia		8,941
10	Erythropoietin AND Dialysis		4,158
11	Erythropoietin AND Haemodialysis		4,162
12	Erythropoietin AND Health Cost		361
13	Erythropoietin AND Treatment Adherence And Compliance		140
14	Erythropoietin AND Patient Satisfaction AND Quality Of Life		20
15	Erythropoietin AND Chronic Kidney Disease AND Dialysis		2,829
16	Erythropoietin AND ESRD AND Dialysis		2,468
TOTAL SEARCH COUNT		41,271	
DUPLICATED ARTICLES		35,419	
TOTAL ARTICLES FILTERED (EXLCUDING DUPLICATES)		5852	

These were then further filtered for articles that were pertinent to the confines of this review by another search carried out using “EndNote X9 ®”, a citation management software, by utilizing a comprehensive list of relevant MeSH terms and regular keywords (see Table 2). Ultimately we were able to narrow down our search to 160 articles that we felt were pertinent to the scope of this review. Out of 160 articles, 38 full-text publications were then chosen for discussion in this literature review, with the merit of selection being individual article relevance and comprehensiveness.

**Table 2:** A comprehensive list of various MeSH terms and regular keywords used for filtering relevant articles after the initial search, using “EndNote X9 ®” ESRD: End-stage renal disease; MeSH: Medical Subject Heading

MeSH Terms	Regular Keywords
Erythropoietin	Chronic renal disease
Erythropoietin / adverse effects	Costs
Erythropoietin / pharmacokinetics	Patient preference
Kidney failure, chronic	Anemia
Chronic kidney disease	ESRD
ESRD	Erythropoietin
Renal dialysis	Convenience
Health cost	Haemodialysis
Epoetin alfa	Chronic kidney disease
Erythropoietin / administration & dosage	Injection
Kidney failure, chronic / complications	Preference
Injections, intravenous	Subcutaneous
Injections, subcutaneous	Intravenous
Quality of life	Haemodialysis
Patient compliance	Administration route
Treatment adherence and compliance	Pain
Patient satisfaction	Efficacy
Patient preference	Safety and tolerance

Discussion

The major factors that govern the route of ESA administration include the patient’s stage of CKD, efficacy considerations, the type of ESA used, dosing frequency, convenience, healthcare costs, and drug safety and tolerability [8,12]. This literature review aims to discuss the existing, relevant literature for these factors with respect to the route of ESA administration and to define areas that need further exploration.

Dosing Frequency

Numerous studies and trials have documented evidence strongly suggestive of the advantages that the SC route of erythropoietin administration has, in terms of requiring a lower dose and frequency of administration, over the IV route (see Table 3)**.**

**Table 3:** A summary of studies comparing dosing frequency between intravenous and subcutaneous routes of administration of erythropoietin HD: Haemodialysis, SC: Subcutaneous, IV: Intravenous, Hb: Haemoglobin, r-HuEPO: Recombinant Human Erythropoietin, ESRD: End-stage Renal Disease.

Serial Number	Author/Year	Study Design	Population	Relevant Conclusive Points
1	Muirhead et al. / 1992 [13]	Clinical Trial	128 adult HD patients selected from five dialysis centers, 45 patients withdrew due to various reasons. 45 patients were in the subcutaneous group (SC) and 38 in the intravenous (IV) group.	Mean dose at stabilization of Hb levels, time to achieve target Hb levels and time to stabilization of Hb levels of rHuEPO were all significantly lower in the SC compared to the IV group.
2	Wright et al. / 2015 [14]	Comparative Study (retrospective cohort)	62,710 adult HD patients enrolled in the Centers for Medicare and Medicaid Services ESRD Clinical Performance Measures Project from 1997 to 2005 were treated with epoetin, of which 57,602 patients received IV and 5108 received SC epoetin.	IV epoetin doses were on average 25% higher than the SC dose for achieving equivalent haemoglobin responses in study patients. Adverse outcomes on follow-up were also found to be significantly more likely in HD patients receiving IV rather than SC epoetin.
3	Vercaigne et al. / 2005 [15]	Clinical Trial	98 adult HD patients already on maintenance SC epoetin therapy enrolled into study and all patients were shifted to IV epoetin simultaneously for the prospective study of anemia. 34 patients withdrew at different stages due to various reasons. 64 patients took part in study.	IV Epoetin requirements increased by 35%, on average, compared to previous SC dosage whilst also resulting in a significantly lower mean Hb level. Similarly at the end of the IV epoetin evaluation period, 52% patients needed more frequent dosing than at the time of SC to IV epoetin conversion.
4	Moist et al. / 2006 [16]	Comparative Study (prospective cohort)	414 adult HD patients participated in this study, which was essentially a wide-scale policy implementation for a change from the maintenance SC epoetin administration route to the IV route. All patients were shifted simultaneously to the IV route. 111 patients withdrew from the study due to various reasons.	The mean weekly, weight-adjusted dose of IV epoetin was found to be 20.2% higher, on average, than the baseline SC dosage. This was most pronounced at 6 and 12 months of follow-up. Patients receiving epoetin 3 times per week increased from 19.6% at baseline, with SC administration, to 79.5% at 12 months of IV epoetin.
5	Galliford et al. / 2005 [17]	Comparative Study (prospective cohort)	86 adult HD patients were studied on a monthly basis for 6 months before and after a change in the route of administration from SC epoetin-alfa to IV epoetin-alfa.	Hb levels fell significantly in the first two months after the switch from SC epoetin-alfa to IV epoetin-alfa administration. This effect was partially offset at 6 months by an increase of IV epoetin-alfa dose by 32% along with an increase in costs.
6	Steffensen et al. / 2011 [18]	Randomized Controlled Trial (Open, multicentre Crossover study)	145 adult HD patients, already on SC Epoetin, were randomized to one of two epoetin treatment groups. The groups either involved treatment with IV epoetin for 4 months followed by SC administration for 4 months or vice versa. Routine iron studies were carried out during study and supplemented as needed.	Provided that iron stores are optimal, there is no significant difference in mean Hb levels and mean EPO doses between IV and SC administration of epoetin-beta.
7	Parker et al. / 1997 [19]	Clinical Trial	44 adult chronic HD patients from a dialysis unit, already on IV Epogen (r-HuEPO, Epoetin-alfa), were selected for this study and subjected to an approved treatment protocol comprised of 3 phases with different routes and doses of Epogen administration. 27 patients completed the protocol over 22 months. 135 “control” subjects were matched during the protocol from another group of dialysis patients refusing to opt for SC r-HuEPO.	The outcomes showed that most chronic, stable HD patients can maintain stable hematocrit and Hb concentrations at once weekly SC EPO doses that are one-third of the required weekly IV dose thereby lending support to its safety and efficacy. Patient safety, serum biochemistry, blood pressure and red blood cell indices were also monitored during the study, with no significant differences in any variable between the control and experimental group.

Drug Safety and Tolerability

As with any other drug, recombinant Human EPO (r-HuEPO) or epoetin carries with it a certain set of side effects. While both IV and SC share some of these adverse effects, the extent and frequency differ between the two (see Table 4). Common to both routes include injection site pain sensation, the development of hypertension, arteriovenous fistulae thrombosis, an increased overall risk of thrombotic and cardiovascular as well as cerebrovascular events, hyperkalemia, depletion of iron stores, flu-like symptoms, a prolonged duration of dialysis and rarely, PRCA and seizures [9,11,12,20].

**Table 4:** A summary of studies comparing erythropoietin safety profile and tolerance between intravenous and subcutaneous administration HD: Haemodialysis, SC: Subcutaneous, IV: Intravenous, Hb: Haemoglobin, rHuEPO: Recombinant Human Erythropoietin, ESRD: End-stage Renal Disease, AE: Adverse events, EPO: Erythropoietin, PRCA: Pure Red Cell Aplasia

Serial Number	Author/Year	Study Design	Population	Relevant Conclusive Points
1	Lee et al. / 2009 [21]	Randomized Controlled Trial	78 adult HD patients were randomly assigned to either receive IV or SC epoetin. The time to Vascular access failure was analysed. Seven patients were withdrawn from evaluation due to various reasons.	Patients in the SC therapy group exhibited a significantly higher rate (12.0%/patient year) of access failure as compared to the IV epoetin group (4.7%/patient year). The study was limited, however, by a small sample size and asymmetry between the two groups.
2	Klinkmann et al. / 1992 [22]	Clinical Trial (Prospective, Multi-center study)	362 adult HD patients from 16 European dialysis centers entered the study with half of the patients receiving r-HuEPO during the first year (first phase) of the trial and then serving as the control group during the second year (second phase) and the other half followed the opposite treatment plan. These patients were monitored for the drug safety of SC administered EPO. A total of 73 patients dropped out from the study.	Adverse events (AE) were recorded in the two groups, as serious and non-serious. AE were higher in the SC therapy group (55.9%) compared to the control group (44.1%), with serious AE being slightly higher in the therapy group. Statistically however, there was no significant difference between the two groups in terms of serious AE like hypertension, loss of vascular site access, respiratory and gastrointestinal system-related issues. Death due to cardiac issues was higher in the control group. NO anti-EPO antibodies were detected in either group. SC EPO administration has demonstrated a better safety profile than IV EPO.
3	Schaller et al. / 1994 [23]	Clinical Trial (Randomized, double-blind, Prospective, Multi-center study)	90 adult HD patients already suffering from ESRD, were enrolled and randomly assigned to 4 different groups, 2 of which comprised of IV EPO therapy and the other 2 SC EPO therapy. The study involved 4 different phases of treatment.	30% of all treated patients exhibited hypertensive reactions. During the Hb and hematocrit correction phase, there were more patients becoming hypertensive with SC EPO therapy than with IV EPO. This was not the case during the maintenance phase of treatment, however, SC EPO-treated patients did not complain about injection site pain and adverse reactions. The relationship between EPO treatment, development of hypertension and route of EPO administration is complex and multifactorial.
4	Kharagjitsingh et al. / 2005 [24]	Multi-center Cohort study	Existing patient data and serum samples from 1677 patients participating in The Netherlands Cooperative Study on the Adequacy of Dialysis-2 (NECOSAD-2) were used in this study. Data was collected at 6-month intervals between April 1997 and September 2002. The study was performed to detect EPO hyporesponsive patients, EPO antibodies and PRCA in dialysis patients.	EPO hyporesponsiveness has numerous causes, prominently infection, inflammation and depletion of iron stores. 57 patients were found to be EPO hyporesponsive, an estimated incidence of 16.7/1000 patient years on EPO while on dialysis. Only one patient among the above 57 patients was found to have clinical PRCA, an estimated incidence of 0.29/1000 patient years on EPO while on dialysis. The incidence of EPO antibodies stood at 1.27/1000 patient-years since the start of dialysis. Out of these 57 patients found to be EPO hyporesponsive, 6 were treated with EPO IV, while all others used EPO SC. anti-EPO antibodies and PRCA remain a rare cause of EPO hyporesponsiveness, though it may be tied to SC EPO administration.
5	Navarro et al. / 1995 [25]	Clinical Trial	13 chronic haemodialysis patients, who remained hypertensive after being on long term (>12 months), thrice weekly, post HD IV rHuEPO therapy were selected for this study, with hypertension being defined as elevated blood pressure that necessitated the use of anti-hypertensive medications. These patients were switched to SC EPO thrice weekly for 6 months, whilst keeping the total weekly SC dose at two-third of the weekly IV rHuEPO dose. Their blood pressure was monitored prior to each HD session. Patient’s hypertensive therapy and red blood cell indices were also analysed regularly.	At the end of the first month of the switch to SC rHuEPO, there was a significant drop in pre-dialysis mean arterial pressure as opposed to baseline pressures, prior to the switch. The number of hypertensive patients reduced from 13 at the time of baseline recording to 8 at the end of the six-month trial. In the remaining 8 hypertensive patients, the severity dropped significantly as was measured by a “therapeutical score” that assessed hypertensive severity from the antihypertensive power of the drugs used to control it. This study shows better control of hypertension with the SC route of administration in ESRD patients and that SC rHuEPO doesn’t prevent hypertension in ESRD patients, rather only reduces its severity compared to the IV route.

Drug-associated Costs

Patients with severe anemia secondary to CKD < Hb 9.0 gm/dl and those with advanced CKD, for example those on regular HD, need prolonged periods of ESA therapy to improve their QoL, to prevent anemia-related symptoms, and to minimize the need for blood transfusion [8]. This can incur significant recurring costs on individuals and on healthcare systems. Dealing with this by employing a cost effective yet efficacious means of ESA therapy is therefore crucial (see Table 5).

**Table 5:** A summary of studies comparing healthcare costs between intravenous and subcutaneous EPO administration HD: Haemodialysis, SC: Subcutaneous, IV: Intravenous, EPO: Erythropoietin, CKD: Chronic Kidney Disease, CAD: Canadian Dollars

Serial Number	Author/date	Study design	Population	Main points
1	Wazny et al. / 2013 [26]	Retrospective Multicenter Study	Patients were chosen from 4 in-centre Haemodialysis Units in Winnipeg, Manitoba, Canada. Patients were treated with Epoetin-alfa in two separate treatment regimes in two separate time periods - each lasting 6 months. 622 individuals were subject to on IV EPO (period 1), and 609 individuals to SC EPO (period 2). Costs were analysed retrospectively from available patient data and monthly inventory billing records.	The switch from IV to SC EPO across 4 haemodialysis units, resulted in a 12.6% dose reduction and saved 98% of the patients receiving SC epoetin alpha, about 1125 USD per person per year.
2	Galliford et al. / 2005 [17]	Comparative Study (prospective cohort)	86 adult HD patients, already on SC EPO-alfa treatment, were switched simultaneously to IV EPO-alfa, at the same weekly dose as their SC administration, for a period of 6 months. Monthly Red cell indices, weekly EPO dosages and other parameters were monitored during the study.	Transitioning from SC to IV EPO alpha in HD patients requires a dose increase of around one-third, possibly resulting in an annual increase in cost of £ 1500 per patient.
3	McFarlane et al. / 2007 [27]	Controlled Clinical Trial	158 adult, chronic, HD patients, already on IV EPO therapy, were studied for 1 year prior to the trial. In the study that spanned 12 months, patients were collectively shifted to SC EPO therapy.	The cost of anemia therapy rose significantly 6 months post-switch to IV EPO therapy. The median rise in costs over the whole 6-month period was estimated at 13.1% (CAD 665/patient-year; p < 0.01).
4	Prasad et al. / 2020 [28]	Retrospective Observational Study	Two hundred and fifteen patients aged more than 18 years, receiving in-center HD for at least 6 months at 4 HD centers. Patients suffering from anemia of CKD requiring epoetin alfa therapy, and on IV epoetin alfa therapy for at least 6 months, were switched to SC EPO-alfa. Data was collected from 6 months prior to 12 months after the switch. Primary outcome was the assessment of epoetin-alfa cost per patient per month before and after the policy change.	Administering epoetin alpha subcutaneously resulted in a dose reduction from IV to SC of 30.51% and 25% reduction in EPO costs, being equally effecting at maintaining Hb levels in patients on HD.

Drug Efficacy

There are various factors that underpin ESA efficacy, i.e., the dose needed to attain a certain target Hb concentration or hematocrit level, which can be adequately summarized under the umbrella of individual ESA pharmacokinetics and pharmacodynamics [12]. Discussed further are factors that have been found relevant to ESA efficacy (see Table 6). Numerous studies have provided support to the SC route of administration due to multiple advantages over the IV route, most notably a lower overall dose to achieve a similar target Hb concentration as well as hematocrit levels and a reduced dosing frequency, i.e., the SC route offers more efficacy for administration of r-HuEPO [13-17,19]. Although the SC route offers a much lower level of bioavailability as compared to the IV route, it results in a significantly longer half-life, attaining peak plasma levels that are substantially lower than the IV route but persist for a much longer period of time. The reasons theorized behind this low bioavailability but a paradoxically prolonged maintenance of modest serum plasma levels can be attributed to a multiple injection site, drug inherent and systemic factors. This persistence and delayed absorption of EPO from SC administration has been pivotal in the explanation for this route’s effectiveness over the IV route. As erythropoiesis is not as dependent on peak plasma EPO levels as it is on the maintenance of EPO levels above a critical threshold for a prolonged time duration, the SC route offers an advantage. IV EPO dosing results in a fall in serum r-HuEPO levels during the interdialytic period and ultimately in the apoptosis of EPO-dependent erythrocyte precursor cells in the bone marrow. SC EPO dosing prevents this apoptosis due to maintenance of plasma EPO levels for a longer duration therefore enabling a more protracted, efficient and effective process of erythropoiesis [29,30].

**Table 6:** A brief summary of studies comparing erythropoietin pharmacokinetics between intravenous and subcutaneous administration HD: Haemodialysis, PD: Peritoneal Dialysis, CAPD: Continuous Ambulatory Peritoneal Dialysis, SC: Subcutaneous, IV: Intravenous, IP: Intra-peritoneal, Hb: Haemoglobin, EPO: Erythropoietin, rHuEPO: Recombinant Human Erythropoietin, ESRD: End-stage Renal Disease, rcEPO: Recombinant Human Erythropoietin, C_max_: Peak serum drug concentration, t_1/2_: drug half-life, S.D.: Standard Deviation

Serial Number	Author / Year	Study Type	Population	Relevant Conclusive points
1	Brockmöller et al. / 1992 [31]	Prospective study	12 adult, chronic, stable HD patients, already under treatment with a thrice weekly IV rcEPO, were subjected to treatment scheme using regimes of IV and SC rcEPO recombinant human EPO (rcEPO) injections in discrete phases to assess pharmacokinetics and therapeutic response to both routes. Serum analyses were carried out at specific time intervals for achieving the goals of this study.	After first dosing with IV EPO, plasma EPO levels were found to have a mean (±S.D.) half-life of 5.4 ± 1.70 hours compared to initial SC EPO administration with a mean(± S.D) absorption time being 22 ± 11 and an average bioavailability of 44% (28-100%). With continuous long-term treatment with IV EPO, elimination half-life reduced by 15% to around 5 hours, possibly a reflection of an increase in hematocrit. The study suggests that the SC route be more effective due to prolonged plasma rcEPO elevation following SC administration, with the exact mechanism being unclear.
2	Nielsen / 1990 [32]	Clinical Trial	Two groups of adult, chronic, and stable HD patients were enrolled. Group 1 was already under maintenance treatment with IV recombinant human EPO (rhEPO) thrice weekly. Group 2 included ESRD patients not previously treated with rhEPO. Both groups were subjected to IV and SC rhEPO administration at different dosages – 50 U/kg for group 1 and 150 U/kg for group 2. Pharmacokinetic studies were then carried out using serum analytics.	After IV rhEPO injections at the lower dose, the mean half-life was found to be 5.4 ± 0.90 hrs, while at the higher dose it was around 7.60 hrs. Peak serum EPO levels (C_max_ ) after IV dosing were found to be 20 times that of SC C_max_. Peak serum EPO levels after SC dosing were reached on an average of 27.3 ± 8.6 hrs. Mean bioavailability was also found to be a meager 14.1% after SC dosing. Despite the data, the protracted maintenance of rhEPO levels after SC administration may be more efficacious than IV dosing though more work is needed in this area and patients with SC administration need to be closely monitored for anti-EPO antibodies.
3	Neumayer et al. / 1989 [33]	Clinical Trial	29 adult, chronic, and stable HD patients, were enrolled and split into 3 groups. Group I comprised of 19 patients who were treated with IV rhEPO initially, then kept on maintenance therapy for 3 months and thereafter 10 patients from this group, making up group II, were subject to another bolus dose of IV rhEPO at the end of these 3 months. Group III was made up of 9 additional patients who were treated with a single SC rhEPO dose. Pharmacokinetic profiles of these two administration routes at different stages of treatment was then assessed using serum studies.	Peak plasma levels after IV dosing were seen within 5 minutes of administration and were not significantly different between Group I and II. IV rhEPO elimination half-life was found to be an average of 8.75 (7.29 - 11.68) hours, in Group I, but fell significantly after 3 months, i.e. in Group II, to 6.80 hours. SC rhEPO peak levels, though 5% that of IV levels, were attained between 18-24 hours after administration, with a mean half-life of 11.2 (7.0-13.9) hrs. SC bioavailability was also low at around 25%. The study questions the benefit of reaching high peak serum levels immediately after IV administration and provides support to a relatively low dose SC administration as mimicking EPO physiological levels in augmenting erythropoiesis.
4	Ateshkadi et al. / 1993 [34]	Clinical Trial	8 stable peritoneal dialysis (PD) patients participated in a randomized, single-dose, three-way cross-over study with Continuous Ambulatory PD (CAPH) being carried throughout the study. Patients were already using EPO or candidates for it. They were given a single average dose of 99.1 U/kg of intraperitoneal (IP), IV, and SC rhEPO. Pharmacokinetics of the three routes were compared using serum analysis studies.	C_max_ for the IP and SC routes are almost identical but only 5% of the IV route. Peak plasma concentrations (C_max_) were attained at a mean of 9.4 ± 1.90 hrs for the IP route, compared to a much slower time for SC, at 17.1 ± 5.0 hours. However, SC bioavailability, 22.81%, was twice that of IP EPO, 11.4%. Compared to the IP route, the SC route had a significantly higher area-under-the-curve (AUC) between 0 and 96 hours after administration. The study also found the potential effect of EPO administration into a “dry” or empty peritoneum for greater efficacy via this route, albeit significantly lesser than the SC route. Administration strategies involving a more prolonged EPO absorption with a relatively low C_max _may enable more efficacy of rhEPO.
5	Macdougall et al. / 1989 [35]	Clinical Trial	8 adult, stable, chronic CAPD patients were enrolled. Each patient was administered intraperitoneal (IP), IV, and SC rhEPO at a set dose for each route. The doses were spaced by 4 weeks.	IV administration exhibited a serum peak level being attained at 15 minutes post administration, with a mean half-life (t_1/2_) of 8.20 (6.20 - 10.20) hrs. IP administration saw C_max _at 12 hours and an average bioavailability of 2.90% (1.2 - 6.8%). C_max _for SC administration was at 18 hours and had a mean bioavailability of 21.5% (11.3 - 36.0%). The study found that t_1/2_ for IV EPO in CAPD patients was not significantly different from those on HD. The findings of the study also suggest that high serum peaks of EPO are of little therapeutic value for effective erythropoiesis. As a result, it suggests that SC route of administration may be more beneficial in both CAPD and HD patients. The bioavailability of this route, however, is governed by a complex interplay of injection site, drug composition and systemic factors.

Route of Administration and Stage of CKD (Non-Dialysis Dependent and Patients on Peritoneal Dialysis vs Haemodialysis Patients)

Non-dialysis CKD patients with preserved GFR, or those undergoing peritoneal dialysis, benefit from SC administration of ESAs, considering that it's least invasive and can be carried out without any monitoring. Furthermore, intraperitoneal administration in patients on continuous ambulatory peritoneal dialysis (CAPD), can dilute ESA concentration, limiting its use [34,35]. The advantage of IV administration lies in the fact that it can be conveniently administered during the process of haemodialysis. Numerous studies have shown that SC doses of ESAs in non-dialysis dependent and patients on peritoneal dialysis, were found to effectively increase Hb concentrations and were well-tolerated and may even be more efficacious than IV EPO formulations in HD patients as well [36,37]. However, more work needs to be done comparing the efficacy of SC versus IV EPO administration in non-dialysis and patients on continuous ambulatory peritoneal dialysis (see Table 7). The use of EPO in HD patients has been covered under other sections.

**Table 7:** A comparison of the different routes of ESA administration with the stage of CKD (Non-dialysis dependent AND Patients on Peritoneal Dialysis vs Haemodialysis patients) HD: Haemodialysis, PD: Peritoneal Dialysis, CAPD: Continuous Ambulatory Peritoneal Dialysis, SC: Subcutaneous, IV: Intravenous, Hb: Haemoglobin, EPO: Erythropoietin, r-HuEPO: Recombinant Human Erythropoietin, ESRD: End-stage Renal Disease, CKD: Chronic Kidney Disease, QoL: Quality of Life

Serial Number	Author/date	Study Design	Population	Main Points
1	Hughes et al. / 1990 [36]	Randomized Controlled Trial	15 adult ESRD patients, on CAPD for the treatment of ESRD were enrolled in this study. They were subjected to treatment with r-HuEPO thrice weekly SC, with two separate target Hb levels in two discrete phases – the correction phase and the maintenance phase.	Thrice weekly administration of r-HuEPO to subjects on peritoneal dialysis effectively corrected renal anemia. A reduction in ineffective erythropoiesis and much more importantly, an increase in erythroid activity was thought to be the major factor in increasing red cell volumes. The findings of this study also suggested that prolonged, moderate increase in serum EPO concentration is more important than a sudden rise in EPO as would be observed with IV EPO administration.
2	Montini et al. / 1993 [37]	Multi-center Study	24 children, suffering from anemia secondary to ESRD and on peritoneal dialysis, aged 3 months to 18 years, were treated with SC r-HuEPO, in varying doses, depending on the Hb levels achieved with each dose.	Eighteen patients experienced increased Hb levels after 24 weeks of treatment from a mean of 6.5 (4.7-7.9) gm/dl to 9.4 ± 1.7 gm/dl. The IV route of administration is less convenient in patients on peritoneal dialysis because of lack of vascular access and difficult self-treatment, while the intraperitoneal route of administration compromises bioavailability significantly.
3	Trivedi and Brooks / 2003 [38]	Comparative Study (retrospective)	31 pre-dialysis CKD patients' medical record was assessed. These patients had been treated with EPO between 1996 and 2001. Various parameters were assessed including renal function, red cell indices, and iron profiles.	The mean hematocrit increased from a baseline value of 28.4 ± 2.7 to 33.6 ± 3.4% after an average of 6 weeks of treatment and to 37.7 ± 4.5% after about 3 months of treatment. By analyzing a variety of other parameters as well, the study importantly concluded that pre-dialysis CKD patients exhibited significant response to EPO therapy without parenteral iron therapy. It was also evident that pre-dialysis CKD patients had lower overall EPO dosage requirements than ESRD patients. However, it is important to note that these findings are similar to the ones in ESRD patients. More work is specifically needed in pre-dialysis patients for analyzing dose requirements among the two routes.
4	Stevens et al. / 1991 [39]	Clinical Trial	Sixteen anemic patients with an Hb < 9 gm/dl, maintained on chronic continuous ambulatory peritoneal dialysis (CAPD), were given SC epoetin-alfa thrice weekly, in two different phases – each with a higher Hb level target than the previous one. The dose of SC EPO was changed periodically, depending upon the results of red-cell indices and target Hb levels.	15 patients responded to treatment with a rise in Hb concentration of more than 2 gm/dl. SC administration was found to be acceptable, convenient more effective in treating anemia in CAPD patients. It was also associated with an improved QoL and can very well be thought of as an optimal route of EPO administration in CAPD patients. Additionally, in the same dialysis unit, it was found that CAPD patients required a lower dose via the SC route than HD patients did via the IV route for maintaining target Hb levels.

Convenience of Drug Administration

Between the two routes, convenience depends on factors like the stage of CKD, the dose and dosage frequency, the type of ESA being used, ease-of-use, the type of dialysis being utilized, the associated healthcare costs, and patient satisfaction.

For non-HD patients, the SC route may be more generally convenient due to the lack of a continuous IV access, the ease of self administration, comparatively lower dosage, less frequent hospital visits, a reduced dosing frequency and ultimately reduced costs. Even in HD patients, the SC route has been tied to similar advantages and may therefore be more beneficial overall as compared to the IV route, despite the obvious convenience that an arteriovenous fistula confers to IV EPO administration [13-17, 19, 26-28, 31-35]. This may be particularly beneficial in low-income countries where affordability and access to newer, longer acting ESAs may be difficult [40]. Evidence lending support to the efficacy, cost effectiveness and safety of the SC route of ESA administration has been presented in earlier sections.

A multicenter study, non-randomized, open-label study conducted by Grzeszczak et al. in 128 stable, chronic PD patients already on once to thrice-weekly SC EPO administration who were enrolled in a study where the effect of shifting them to once-weekly and once-fortnightly administration of SC Epoetin-beta in maintaining their Hb concentrations, was investigated. The findings concluded that shifting patients to SC Epoetin-beta once-weekly did not result in a significant change in mean Hb levels over a period of 25 weeks. In the once-fortnightly group, the dose needed to be increased slightly and even then, more than 50% of patients could still be maintained on baseline EPO-beta doses or lower. This study paves way for a means of ESA administration that could result in greater convenience, compliance, patient satisfaction, reduced dosage frequency and greater cost savings [41].

The convenience of use for the IV formulation in HD has its possible roots in the preference for its use by HD Staff. This may be due to the routine use of the IV route by HD staff [16] as well as the issue of pain or ‘stinging’ or discomfort at the injection site associated with the initial use of SC epoetin-alfa which some studies have cited in the past [42]. The KDIGO guidelines have also cited ‘pain’ secondary to SC administration, in terms of using short-acting ESAs, as a reason to prefer IV EPO administration, referring to the results of a single centre trial of 30 patients [8, 26]. In a randomized, un-blinded trial carried out by Kaufman et al. among 208 patients, 86% of the 107 candidates who received SC epoetin injections reported experiencing pain as none to mild [43]. Similar findings were found in a multi-center randomized, double-blind, prospective study among 90 ESRD patients already on HD, who were subjected to different regimes of IV and SC EPO treatment. None of the patients treated SC complained of injection site pain nor were there any identifiable local adverse reactions [23]. Nonetheless the pain reported in previous studies has mainly been tied to the citrate component of the epoetin-alfa buffered solution that is administered SC to patients [44]. However, it has largely been controlled by replacement of the citrate preservative with Benzyl Alcohol saline and other newly developed stabilizer solutions, using large gauge needles, and smaller volume doses, all of which were highly effective in reducing pain whilst maintaining drug efficacy [43, 45, 46].

Type of ESA Formulation Used

ESA can generally be classified as short-acting or long-acting. The former usually refers to the 1st generation ESAs like Epoetin-alfa and beta while the latter normally refers to 2nd generation ESAs and beyond, including formulations like Darbepoetin-alfa and continuous erythropoietin receptor activator (C.E.R.A) [4]. The choice of ESA depends on factors like drug availability, affordability, patient preference, and local policies. Each of these agents also has its own unique pharmacokinetic and pharmacodynamic profiles [4, 8]. In this review we shall only discuss common ESA types from the first and second generations (see Table 8).

**Table 8:** A general comparison of some commonly used 1st and 2nd Generation Erythropoiesis Stimulating Agents, based on previous clinical trails HD: Haemodialysis, SC: Subcutaneous, IV: Intravenous, Hb: Haemoglobin, EPO: Erythropoietin, rHuEPO: Recombinant Human Erythropoietin, ESRD: End-stage Renal Disease, rcEPO: Recombinant Human Erythropoietin, t_1/2_: drug half-life, ESA: Erythropoiesis Stimulating Agent

Serial Number	Author / Year	Study Type	Population	Relevant Conclusive Points
1	Bernieh et al. / 2014 [47]	Randomized Controlled Trial	139 adult, chronic HD patients on Eprex (epoetin-alfa) for the last 3 months were randomized to three groups: Group-A-1 receiving long-acting ESA Darbepoetin-alfa once weekly, Group-A-2 receiving Darbepoetin-alfa once in two weeks and Group-B representing patients continued on Eprex treatment.	Darbepoetin alfa given weekly or once in two weeks proved to be more effective (64.8% of all patients in Group-A) in achieving target Hb levels than epoetin-alfa (59.7% of all patients in Group-B), with significantly less dose changes and vascular site thrombosis in the former group compared to the latter.
2	Sinha et al. / 2019 [48]	Clinical Trial (a prospective phase III, randomized, open label, two-arm, parallel group, multi-center study)	Adult anemic, chronic HD dependent patients already on EPO-alfa, were started on an EPO regime comprised of Darbepoetin-alfa, in two phases: correction phase (12-24 weeks) and maintenance phase (24-36 weeks). 126 patients in both phases were randomized in 1:1 ratio to receive either Darbepoetin-alfa once weekly or EPO-alfa thrice weekly.	Darbepoetin-alfa, when administered at a reduced frequency and comparatively lower dose, is similar to epoetin-alfa in terms of achieving and maintaining target Hb levels. Darbepoetin-alfa also has a slightly better safety profile. This may pave way for achieving methods of treating renal anemia that are simpler and with mutual benefits for patients and healthcare staff.
3	Bommer et al. / 2008 [49]	Randomized Controlled Trial (prospective, randomized, multicentre study)	126 adult, chronic, stable HD patients at 9 German dialysis centres, already on Darbepoetin-alfa for at least 6 months, were randomized to either continue with their previous treatment plan or to IV Darbepoetin-alfa in the same dose and frequency.	As opposed to the significant changes in dosage, dose frequency and incurred costs that one encounters in changing EPO-alfa from SC to IV administration, Darbepoetin-alfa does not exhibit such a trend. Instead, it exhibits a similar pharmacokinetic profile for both the IV and SC route. Therefore it presents a better cost-benefit profile and an easier to use choice among ESAs.
4	Allon et al. / 2002 [50]	Clinical Trial (multicenter, randomized, open-label study)	47 adult, chronic, stable HD patients, already being treated with IV epoetin since at least 2 months, were randomized to 3 treatment groups for 6 months – one to receive IV Darbepoetin alfa once weekly, one to receive IV Darbepoetin alfa thrice weekly and the last to receive IV epoetin alfa thrice weekly.	IV Darbepoetin alfa has a t_1/2_ that is 2 - 3 times longer and a clearance 4 times slower than that of epoetin alfa. The data gathered showed no changes in Darbepoetin pharmacokinetics with dose frequency or amount, as compared to epoetin alfa. The results lend support to the use of Darbepoetin alfa by healthcare staff owing to its flexible safety and efficacy at lower and less frequent doses compared to epoetin.

Limitations

As no review or study is perfect, this literature review has an important set of limitation: papers were excluded if they were published in a language other than English, study types that met our exclusion criteria like commentaries or animal-based studies were excluded, our focus was mainly limited to studies based on adult CKD patients, and we mainly covered 1st and 2nd generation ESAs in our review. Additionally, the majority of studies in anemic CKD patients included in this review pertain to HD dependent ESRD patients. While significant efforts were made to review a broad range of publications from different years, it is still pertinent to mention the selection bias that may be present in this review with regards to the articles chosen for review and their relevance. Future research work that corrects these limitations may very well lead to different outcomes or impressions on the reader.

## Conclusions

The studies discussed in this literature review are suggestive of greater efficacy and benefits via the SC route of EPO administration over the IV route in most cases of chronic kidney disease, keeping in view the factors mentioned earlier - drug costs, efficacy, convenience, dosage frequency, stage of CKD, type of ESA used, and drug safety and tolerability. More work, however, is definitely needed in elucidating the individual effects of each of these factors among the two routes of administration, using multi-center randomized trials with much larger sample sizes, especially on the use of ESAs according to the stage of CKD and holistic comparisons of long acting against short acting ESAs, whilst measuring secondary outcomes like patient satisfaction and convenience of use. This may very well help formulate more practical guidelines, help improve the efficiency of ESA use, ensure cost saving, improve quality of care for patients and provide clinicians with better insight during decision making.
